# Evaluation of the BDCA2-DTR Transgenic Mouse Model in Chronic and Acute Inflammation

**DOI:** 10.1371/journal.pone.0134176

**Published:** 2015-08-07

**Authors:** Manuela Mandl, Maik Drechsler, Yvonne Jansen, Carlos Neideck, Heidi Noels, Alexander Faussner, Oliver Soehnlein, Christian Weber, Yvonne Döring

**Affiliations:** 1 Institute for Cardiovascular Prevention, Ludwig-Maximilians-University Munich, Munich, Germany; 2 Academic Medical Center, Department of Pathology, Amsterdam University, Amsterdam, the Netherlands; 3 DZHK (German Centre for Cardiovascular Research), partner site Munich Heart Alliance, Munich, Germany; 4 Institute for Molecular Cardiovascular Research, RWTH Aachen University Aachen, Aachen, Germany; University of Amsterdam Academic Medical Center, NETHERLANDS

## Abstract

**Background and Aims:**

Plasmacytoid dendritic cells (pDCs) are a small subset of dendritic cells and the main producers of type I interferons. Besides their contribution to tolerance, they are known to be involved in autoimmune diseases and have recently been implicated in atherosclerosis. However, their precise involvement, particularly in advanced lesion development, remains elusive. Hence, we investigated the role of pDCs in atherogenesis vs atheroprogression by specifically depleting this cell population using the BDCA2-DTR mouse model bred to Apolipoprotein E (*Apoe* ^*-/-*^) deficient mice.

**Methods and Results:**

Our results revealed that continuous diphtheria toxin-induced pDC depletion in *Apoe* ^*-/-*^ *BDCA2-DTR* mice receiving a high-fat diet (HFD) for 4 weeks did not alter lesion size or composition. Instead, these mice displayed increased B cell numbers and altered levels of inflammatory cytokines. Analysis of depletion efficiency showed that complete pDC depletion could only be sustained for one week and reoccurring pDCs sorted after 4 weeks did not express DTR anymore. Consequently, we analyzed lesion development in a model of partial carotid ligation, inducing established lesions after 5 weeks of HFD feeding, and only depleted pDCs during the last week of 5 weeks HFD feeding. Despite short-term, but efficient pDC depletion, we observed no differences in atherosclerotic lesion development, but changes in inflammatory cytokine titers. To assure the functionality of the BDCA2-DTR model in acute settings, we additionally examined the effect of pDC depletion in an indirect acute lung injury (iALI) model. This time, efficient pDC depletion resulted in a significantly reduced macrophage and neutrophil accumulation in the lung 12 hours after LPS challenge, underlining a pro-inflammatory role of pDCs in the innate immune response in iALI.

**Conclusion:**

Taken together, the BDCA2-DTR mouse model only allows efficient pDC depletion for one week, which subsequently restricts its usability to more acute but not chronic inflammatory disease models.

## Introduction

Plasmacytoid dendritic cells (pDCs) are a scarce subset of bone marrow-derived dendritic cells (DCs), known to produce large amounts of type I interferons (IFN-I) in response to mainly viral pathogens. Recognition of pathogen-associated molecular patterns (PAMPs) by pDCs is predominantly mediated by the endosomal Toll-like receptors TLR7 and TLR9 [1]. Further, pDCs are involved in a variety of other functions including the support of T cell survival, B cell differentiation [2], conventional DC (cDC) activation, and T cell-mediated immune responses during chronic infection [3]. In addition, they exert tolerogenic functions e.g. via indoleamine-pyrrole 2,3-dioxygenase or granzyme B[4], but also play an important role in the pathophysiology of different autoimmune diseases like psoriasis and systemic lupus erythematosus (SLE)[5].

More recently pDCs have also gained attention in cardiovascular diseases (CVD) and atherosclerosis. [6, 7] Atherosclerosis, as the underlying pathomechanism of CVD, is triggered by endothelial dysfunction leading to an accumulation of leukocytes and lipids in the arterial intima, ultimately resulting in atherosclerotic lesion formation and subsequent arterial lumen narrowing. [8] PDCs have been identified in both human and mouse lesions, where they were located in unstable plaque regions close to T cells.[9–12] Further, modified lipoproteins like oxidized low-density lipoprotein have been suggested to activate pDCs in vascular inflammation.[13] In line, danger-associated molecular patterns such as self-nucleic acids, antimicrobial peptides or complexes of both were shown to induce pDC-triggered pro-inflammatory immune responses in atherogenesis and inflammation.[11, 14, 15] Especially the strong IFN-I response of activated pDCs has been implicated in driving atherogenesis by inducing endothelial cell adhesion molecule expression as well as through leukocyte attraction and activation. [11, 16–18] In line with these findings, pDC depletion reduced plaque burden in diet-induced atherosclerosis. [14, 19, 20] However, one study reported a protective role of pDCs in atherosclerosis by dampening T-cell proliferation and activity in peripheral lymphoid tissue.[21] In general, all these studies only addressed the role of pDCs in atherogenesis, but do not detail on their role in atheroprogression.

We and others could show that pDC depletion with a pDC-specific antibody significantly reduced lesion burden in atherosclerosis-prone *Apoe* ^*-/-*^ mice after 4 weeks of HFD [14, 19]. Yet, atherosclerosis is a chronic inflammatory disease evolving over several months (mouse models) or years (human subjects). Long term administration of a depletion antibody may come along with severe side effects, which dramatically reduce the specificity and efficacy of the antibody [22]. To overcome this hurdle and to gain further insight in the role of pDCs in developed lesions we wanted to take advantage of the BDCA2-DTR model, which allows for specific depletion of pDCs by administration of diphtheria toxin (DT) [23].

## Materials and Methods

### Mice

Blood dendritic cell antigen 2-diphtheria toxin receptor transgenic (BDCA2-DTR) mice were bred in the local animal facility and fed a normal chow diet. Experimental mice were sex- and age- matching. *BDCA2-DTR* mice were generated for selective depletion of pDCs *in vivo* by expression DTR under control of the human, pDC-specific BDCA2 gene [23]. Further, *BDCA2-DTR* mice were crossed with Apolipoprotein E deficient (*Apoe* ^-/-^) mice to generate *Apoe* ^*-/-*^ *BDCA2-DTR* mice. Female *Apoe* ^*-/-*^ and *Apoe* ^*-/-*^ *BDCA2-DTR* were fed a high-fat diet (HFD) containing 21% fat and 0.15% cholesterol (Sniff) for 4 weeks (atherosclerosis) or 5 weeks (partial carotid artery ligation) starting at 8 weeks of age. All mouse strains were on C57Bl/6 background. All animal experiments were approved by local authorities (Regierung von Oberbayern, Sachgebiet 54, Germany) and complied with the German animal protection law. All surgery was performed under ketamine/xylazin or medetomidin/midazolam/fentanyl anesthesia.

### *In vivo* depletion of plasmacytoid dendritic cells

For the specific depletion of pDCs, *Apoe* ^*-/-*^ control and *Apoe* ^*-/-*^ *BDCA2-DTR* mice were weighed before the experiment and treated with 0.01 mg/kg body weight diphtheria toxin (DT) per mouse (Sigma-Aldrich). DT was administered 3 times weekly by intraperitoneal injection during 4 weeks of HFD feeding. For the partial ligation of the left carotid artery the *Apoe* ^*-/-*^ and *Apoe* ^*-/-*^ *BDCA2-DTR* mice were fed 5 weeks HFD and pDCs were depleted by DT injection during the last week of HFD. For indirect acute lung injury (iALI), pDCs were depleted with one single DT injection 24 hours before euthanasia and 12 hours before LPS administration. The depletion efficiency was confirmed by flow cytometry analysis of pDC fractions (B220^+^mPDCA-1^+^SiglecH-440c^+^) among CD45^+^ leukocytes in bone marrow, spleen and blood.

### Functional analysis of plasmacytoid dendritic cells

For functional analysis of pDCs (Ifn-α production and costimulatory molecule upregulation), bones (femur, tibia, collarbone) of *Apoe* ^*-/-*^ control and *Apoe* ^*-/-*^ *BDCA2-DTR* mice were harvested and flushed with Hank’s medium (Hanks’ Balanced Salt Solution + 0.3 mmol/l EDTA + 0.1% BSA). Cell suspensions were treated and stained according to FACS protocol with following antibody cocktail: anti-CD45 (eBioscience, clone 30-F11), anti-B220 (eBioscience, clone RA3-6B2) and anti-SiglecH (eBioscience, clone eBio440c). After cell sorting, sorted pDCs were cultured in 24 well flat-bottom plates (1x10^5^ cells/well) in RPMI1640 Medium with L-Glutamine (Gibco by life technologies) and 1% Penicillin/Streptavidin with or without 5 μg/ml CpG oligodeoxynucleotides (ODN 1585, InvivoGen). After incubating pDCs 12 hours at 37°C, cell supernatants were used for Ifn-α ELISA and costimulatory molecule upregulation was measured with FACS using anti-CD86 (eBioscience, clone GL1) and anti-MHC class II (BD Pharmingen, clone 2G9) antibodies. To analyze the expression of DTR on pDCs, splenic pDCs of *Apoe* ^*-/-*^ control and *Apoe* ^*-/-*^ *BDCA2-DTR* mice were stained and measured according to the FACS protocol with an anti-hDTR antibody (human HB-EGF, clone #125923, R&D Systems).

### LPS-induced indirect acute lung injury (iALI)

iALI was induced in *Apoe* ^*-/-*^ *BDCA2-DTR* mice, 8 weeks of age, by intravenous injection of lipopolysaccharides (LPS) from Salmonella enterica (Sigma-Aldrich). PDCs were depleted by intraperitoneal injection of DT (see in vivo depletion of pDCs). LPS (100 μg/mouse) was dissolved in Phosphate Buffered Saline (PBS) (Gibco by Life technologies) and administered by tail vein injection 12 hours after pDC depletion. 12 hours after LPS application, the mice were euthanized. The lungs were flushed with 5x0.5 ml PBS to obtain the broncheoalveolar lavage fluid (BALF). The BALF was centrifuged at 300 g for 5 minutes and the cell pellet was stained with specific antibodies for further flow cytometry analysis. The lungs were removed, shredded and digested for 2 hours at 37°C with liberase (25 mg/ml) (Roche Diagnostics). The digested lungs were passed through a 50 μm cell strainer (CellTrics, Partec) and after 5 minutes of centrifugation at 300 g the single cell suspension was stained with specific antibodies for flow cytometry analysis.

### Partial carotid artery ligation

Partial ligation of the left carotid artery was performed as previously described [24]. *Apoe* ^*-/-*^ control and *Apoe* ^*-/-*^ *BDCA2-DTR* mice were fed a high-fat diet for 5 weeks after ligating the left carotid artery and pDCs were depleted by triple DT administration in the last week before euthanasia (see in vivo depletion of pDCs).

### Lipids and atherosclerotic lesion development

Cholesterol and triglyceride levels in mouse EDTA plasma were quantified using enzymatic assays (c.f.a.s. cobas, Roche Diagnostics) according to the manufacturer’s protocol. Leukocyte counts were determined by routine laboratory assays. The extent of atherosclerotic lesion in aortic roots was assessed by staining aortic root cryosections for lipid depositions with Oil Red O. In brief, the hearts with aortic roots or the left carotid arteries were embedded and frozen in Tissue-Tek (Sacura) for cryosectioning. Oil Red O^+^ atherosclerotic lesions were quantified in 4 μm tranverse sections and averages were calculated from 3 sections. Lesion development in the left common carotid artery (partial carotid artery ligation) was quantified by Hematoxylin and Eosin (H&E) staining of 4 μm cryosections. The sections were quantified using a Leica DMRBE microscope and computerized image analysis (Diskus Software) and Leica Qwin Imaging software (Leica Ltd.).

### Immunohistochemistry

The content of macrophages in aortic roots was determined by immunohistochemical staining of aortic root sections. Appropriate IgG antibodies were used as isotype controls. Aortic root cryosections were stained with an anti-Mac2 antibody (Cedarline). After incubation with a secondary FITC-conjugated antibody (Sigma) for 30 minutes at room temperature, the stained slides were counter-stained with 4',6-Diamidino-2-phenylindol (DAPI) for nuclei and embedded with VectaShield Hard Set Mounting Medium (Vector laboratories). The sections were analyzed using a Leica DM4000B LED fluorescence microscope and charge-coupled device (CCD) camera.

### Histopathological analysis

Lungs of pDC depleted or non-depleted control mice were dissected, fixed in 4% Paraformaldehyde (Sigma-Aldrich) and embedded in paraffin. Paraffin-embedded lungs were cut at the microtome (4 μm) and the sections were stained with Hematoxylin and Eosin (H&E). The stained lung sections were histologically examined at a Leica DMRBE microscope.

### Flow cytometry

Whole blood obtained from the retro-orbital plexus of *Apoe* ^*-/-*^ control or *Apoe* ^*-/-*^ *BDCA2-DTR* mice was EDTA-buffered and subjected to red-blood-cell lysis. Bone marrow cells were harvested by flushing femurs with Hank’s Medium (Hanks’ Balanced Salt Solution + 0.3 mmol/l EDTA + 0.1% BSA) (Gibco by life technologies). Spleen and lymph nodes were mechanically disaggregated, flushed with Hank’s Medium and passed through a 30 μm cell strainer (CellTrics, Partec) to obtain single cell suspensions. All single cell suspensions were centrifuged for 5 minutes at 300 g and cell pellets were stained with different antibody cocktails for FACS analysis. Cell populations were discriminated by the following antibody cocktail: anti-CD45 (eBioscience, clone 30-F11), anti-CD115 (eBioscience, clone AFS98), anti-Gr1 (Biolegend, clone RB6-8C5), anti-CD11b (eBioscience, clone M1/70), anti-F4/80 (eBioscience, clone BM8), anti-B220 (eBioscience, clone RA3-6B2) anti-CD3 (eBioscience, clone 145-2C11), anti-mPDCA-1 (Miltenyi Biotec), anti-SiglecH 440c (eBioscience, clone eBio440c), anti-CD11c (BD Pharmingen, clone HL3) and anti-MHC class II (BD Pharmingen, clone 2G9). Leukocyte subsets were defined using FlowJo software: neutrophils (CD45^+^CD115^-^Gr1^high^), monocytes (CD45^+^CD11b^+^CD115^+^), macrophages (CD45^+^F4/80^+^), lymphocytes (CD45^+^CD3^+^ and CD45^+^B220^+^), plasmacytoid dendritic cells (CD45^+^B220^+^mPDCA-1^+^440c^+^) and conventional dendritic cells (CD45^+^CD11c^+^MHCII^+^). Flow cytometry was conducted at a FACS Canto (BD Biosciences).

### Cytokine analysis

Tnf-α, Cxcl1 and Il-6 levels in mouse plasma samples were measured using the ProcartaPlexTM Multiplex Immunoassay technology from Affymetrix (Affymetrix, eBioscience), according to the manufacturer’s protocol. Ifn-α levels from pDC culture supernatants were analyzed according to the manufacturer’s protocol with a VeriKine mouse Interferon Alpha ELISA Kit (pbl Assay Science).

### Statistics

All data are expressed as mean±SD. Statistical calculations were performed using GraphPad Prism 5 (GraphPad Software Inc.). Unpaired Student’s t-test with Welch’s correction or Mann-Whitney tests were used, as appropriate. P-values <0.05 were considered as being statistically significant.

## Results

### The *Apoe* ^*-/-*^ *BDCA2-DTR* mouse model enables efficient pDC depletion using DT treatment in a one-week experimental setup

**Fig 1:**
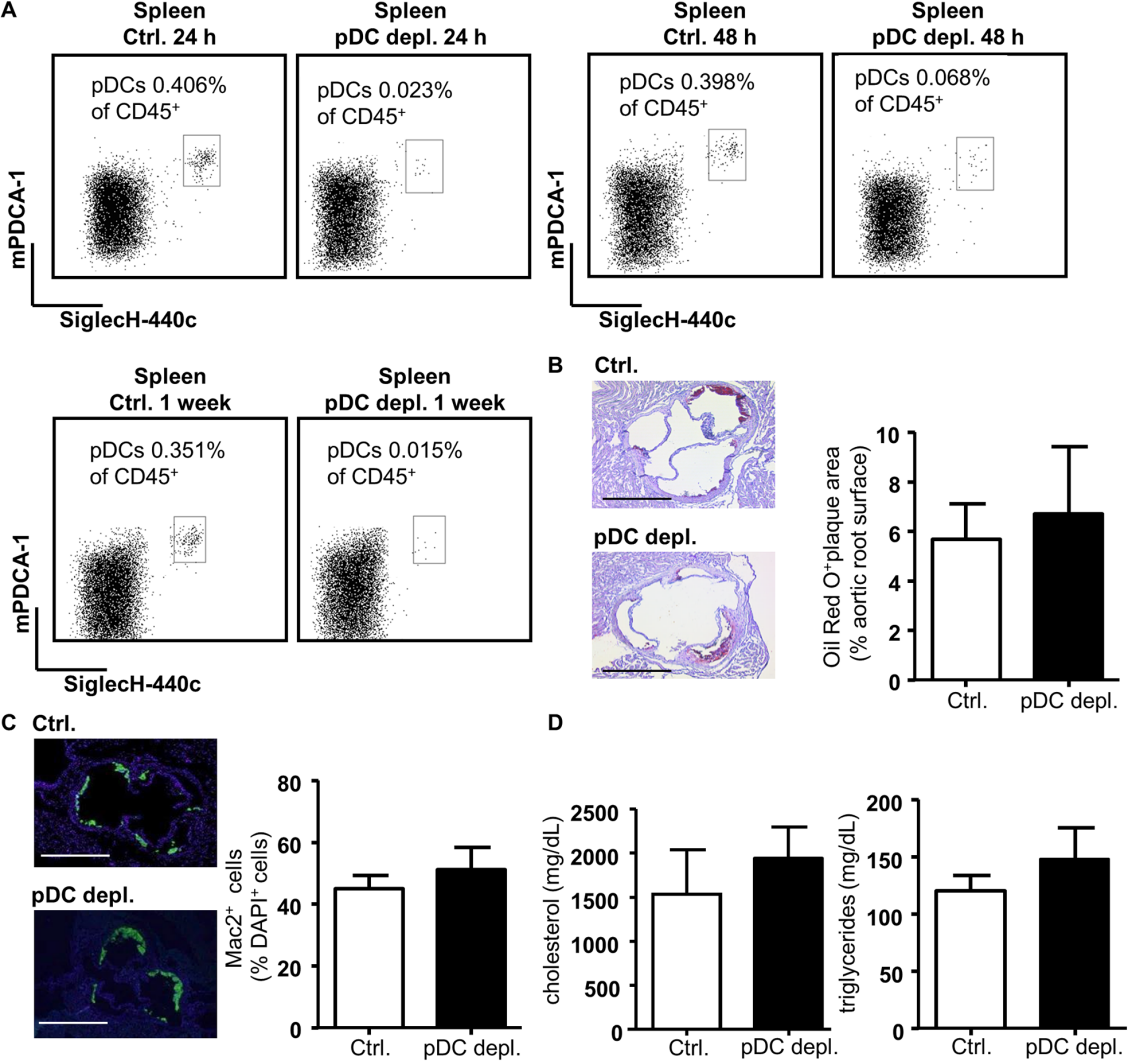
DT induced pDC depletion does not influence atherosclerotic lesion size, lesional macrophage numbers and plasma lipid levels in *Apoe* ^*-/-*^ *BDCA2-DTR* mice after 4 weeks of HFD. (A) Depletion efficiency of pDCs after i.p. DT administration in *Apoe* ^*−/−*^ mice (control) compared to *Apoe* ^*−/−*^ *BDCA2-DTR* mice (pDC depleted). Representative dot plots show B220^+^mPDCA-1^+^440c^+^ pDC populations as percentage of CD45^+^ cells in spleen after 24 and 48 hours (one single i.p. injection) and one week (3 times i.p. injection) after DT administration. (B) Quantification of atherosclerotic lesion size with Oil Red O^+^ area (% of aortic root surface) and (C) Lesional MAC2^+^ macrophages (% of DAPI^+^ cells) in aortic root cryosections of *Apoe* ^*−/−*^ and *Apoe* ^*−/−*^ *BDCA2-DTR* mice after 4 weeks of HFD (scale bar = 500 μm). (D) Plasma cholesterol and triglycerides levels (mg/dL) of *Apoe* ^*−/−*^ and *Apoe* ^*−/−*^ *BDCA2-DTR* mice after 4 weeks of HFD. Graphs represent the mean±SD; n = 6 to n = 10.

As leukocytes crucially affect atherosclerosis, we first compared leukocyte profiles of *Apoe* ^*-/-*^, *Apoe* ^*-/-*^ *BDCA2-DTR* and C57Bl/6 (Bl6) mice to examine potential inherent effects of BDCA2-DTR transgene expression on leukocyte subpopulations. Flow cytometry analysis of blood leukocytes, myeloid cells and lymphocytes did not reveal any significant differences (Fig A in S1 file). Next, we investigated whether i.p. injection of DT could induce efficient pDC depletion for 24 h, 48 h and 1 week. A single dose of DT (0.01 mg/kg i.p.) was injected in *Apoe* ^*-/-*^ and *Apoe* ^*-/-*^ *BDCA2-DTR* mice and pDC numbers were evaluated in spleen after 24 h and 48 h. For analysis of pDC depletion efficiency for one week DT was administered 3 times a week. Subsequent flow cytometry analysis of spleen cells confirmed efficient pDC depletion at each of these time points (Fig 1A). In conclusion, the Apoe^-/-^ BDCA2-DTR mouse model enables efficient pDC depletion using a triple injection of DT in a one-week experimental setup.

### Four weeks DT treatment of *Apoe* ^*-/-*^ *BDCA2-DTR* mice under high-fat diet cannot sustain pDC depletion and does not impact on atherogenesis

Based on the latter and before using the BDCA2-DTR mouse model in long-time atherosclerosis studies, we first evaluated the effect of 4 weeks pDC depletion on atherogenesis to confirm previous studies applying a depletion antibody.[14, 19] *Apoe* ^*-/-*^ and *Apoe* ^*-/-*^ *BDCA2-DTR* mice were fed a HFD while injected with DT 3 times per week over 4 weeks. After 4 weeks atherosclerotic lesion size, composition and plasma lipid levels were evaluated. No differences were detected in aortic root plaque area (Fig 1B) or in the percentage of lesional macrophages (Fig 1C). Further, cholesterol and triglyceride levels in blood were not altered between the two groups (Fig 1D).

**Fig 2:**
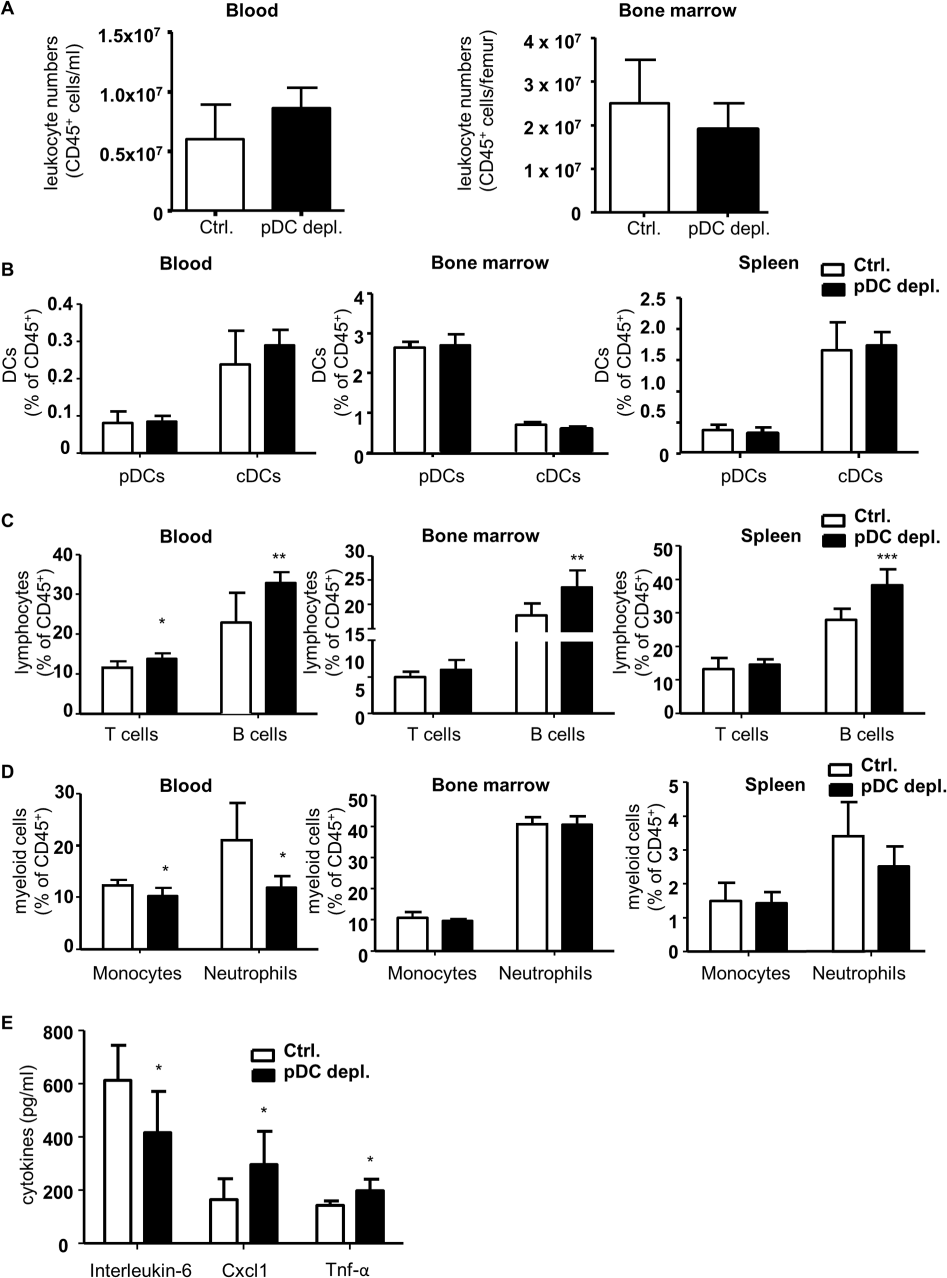
Four weeks DT treatment of *Apoe* ^*−/−*^ *BDCA2-DTR* mice does not alter pDC or CD45^+^ leukocyte numbers, but increases B cells and alters cytokine levels after 4 weeks HFD. (A) Absolute numbers of CD45^+^ leukocytes after 4 weeks HFD in *Apoe* ^*−/−*^ (control) and *Apoe* ^*−/−*^ *BDCA2-DTR* mice (pDC depleted) and in blood (cells/ml) and bone marrow (cells/femur). (B-D) Flow cytometry analysis of (B) dendritic cells (pDCs and cDCs), (C) lymphocytes (T and B cells) and (D) myeloid cells (neutrophils and monocytes) in blood, bone marrow and spleen in *Apoe* ^*−/−*^ and *Apoe* ^*−/−*^ *BDCA2-DTR* mice after 4 weeks of HFD and DT administration. Gr1^+^CD115^-^ neutrophils, CD11b^+^CD115^+^ monocytes, CD3^+^ T cells, B220^+^ B cells, CD11c^+^MHCII^+^ cDCs and B220^+^mPDCA-1^+^440c^+^ pDCs are shown as percentage of CD45^+^ leukocytes. (E) Plasma cytokine levels of Il-6, Cxcl1 and Tnf-α (pg/ml). Graphs represent the mean±SD; n = 6 to n = 10. Mann-Whitney test *P*<0.05 **P<0.01 ***P<0.001.

In parallel we conducted flow cytometry analysis to analyze effects on total leukocytes and leukocyte subsets in blood, bone marrow, spleen and lymph nodes. Total leukocyte numbers in blood and bone marrow did not differ between the two groups (Fig 2A). Furthermore, cDC and to our surprise pDC fractions in blood, bone marrow, spleen and lymph nodes were not altered between both groups after 4 weeks of DT treatment (Fig 2B and Fig Ba in S1 file). In contrast, B cells were significantly increased in all organs (Fig 2C and Fig Bb in S1 file.), and T cells were considerably enhanced in blood of the pDC depleted mice, but not affected in bone marrow, spleen and lymph node (Fig 2C and Fig Bb in S1 file). Instead, monocytes and neutrophils were significantly diminished in blood, but not in bone marrow and spleen in *Apoe* ^*-/-*^ *BDCA2-DTR* mice (Fig 2D). The corresponding cytokine profile in plasma showed a significant decrease of Il-6, but an increase of Tnf-α and Cxcl1 titers in the pDC depleted group (Fig 2E). Levels of Ifn-α and Ccl2 were not altered (Fig Ga in S1 file). Taken together, these data reveal complex changes in leukocyte distribution and inflammatory cytokine levels in *Apoe* ^*-/-*^ *BDCA2-DTR* mice vs *Apoe* ^*-/-*^ controls after 4 weeks of DT treatment. Furthermore, pDC depletion could not be sustained over the 4 weeks study time. Together, the disturbed inflammatory profile combined with insufficient pDC depletion over the entire study period did not allow to examine any intrinsic pDC impact on atherosclerotic lesion development.

### *Apoe* ^*-/-*^ *BDCA2-DTR* mice do not allow continuous pDC depletion for more than one week

**Fig 3:**
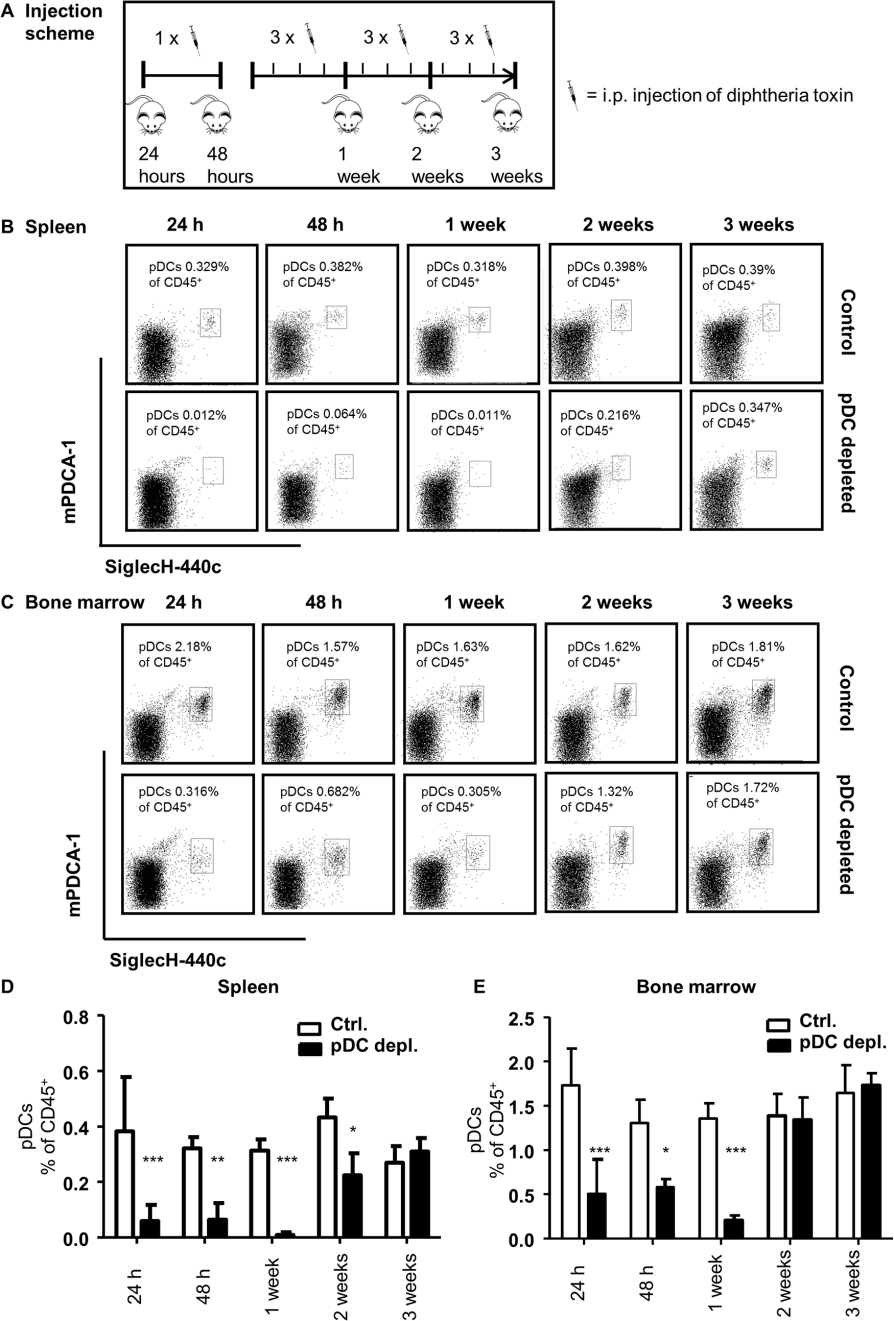
Repeated DT administration efficiently depletes pDCs in spleen and bone marrow up to 1 week. (A) Injection scheme of DT administration. DT was injected once for 24 and 48 hours, 3 times for 1 week and 3 times weekly during 2 and 3 weeks. (B-E) Evaluation of pDC depletion efficiency for different time points: 24 hours, 48 hours, 1 week, 2 weeks and 3 weeks in *Apoe* ^*-/-*^ (control) and *Apoe* ^*-/-*^ *BDCA2-DTR* mice (pDC depleted) after i.p. DT administration. Representative dot plots of pDC numbers in (B) spleen and (C) bone marrow and quantification of pDCs in (D) spleen and (E) bone marrow are depicted. PDCs are shown as CD45^+^B220^+^mPDCA1^+^440c^+^ cells in % of CD45^+^ leukocytes. Graphs represent mean±SD; n = 3 to n = 5. Mann-Whitney test *P*<0.05 **P<0.01 ***P<0.001.

Although reported otherwise [23, 25, 26], our results indicate that long term depletion of pDCs cannot be continued over extended time periods in *BDCA2-DTR* mice. To further investigate this issue we followed up the depletion efficiency for three weeks in blood, spleen, bone marrow and axillary lymph nodes of *Apoe* ^*-/-*^ and *Apoe* ^*-/-*^ *BDCA2-DTR* mice on chow diet. Injecting DT three times weekly for three weeks (Fig 3A), we did see an efficient depletion of pDCs in blood, spleen, lymph node and bone marrow after 24 h, 48 h and 1 week (Fig 3B–3E and Fig CA and CB in S1 file). However, after two weeks pDC numbers were already back to control level in blood and bone marrow (Fig Ca in S1 file and Fig 3C and 3E). Furthermore, although pDC numbers in spleen and lymph node were still reduced after 2 weeks, efficiency of pDC depletion was much lower compared to 1 week DT treatment (Fig 3B and 3D and Fig Cb in S1 file). Three weeks after starting with the depletion of pDCs by repetitive DT injections no significant differences could be detected any longer in any of the four organs investigated (Fig 3 and Fig C in S1 file). This corresponds with our previous observation that pDC levels were not altered in *Apoe* ^*-/-*^ vs *Apoe* ^*-/-*^ *BDCA2-DTR* mice after 4 weeks of DT treatment under HFD, as shown in Fig 2 and Fig B in S1 File. In conclusion, our data show that *Apoe* ^*-/-*^ *BDCA2-DTR* mice do not allow continuous pDC depletion for more than one week.

### The BDCA2-DTR transgene does not influence pDC function in steady state and after 4 weeks HFD with DT treatment

**Fig 4:**
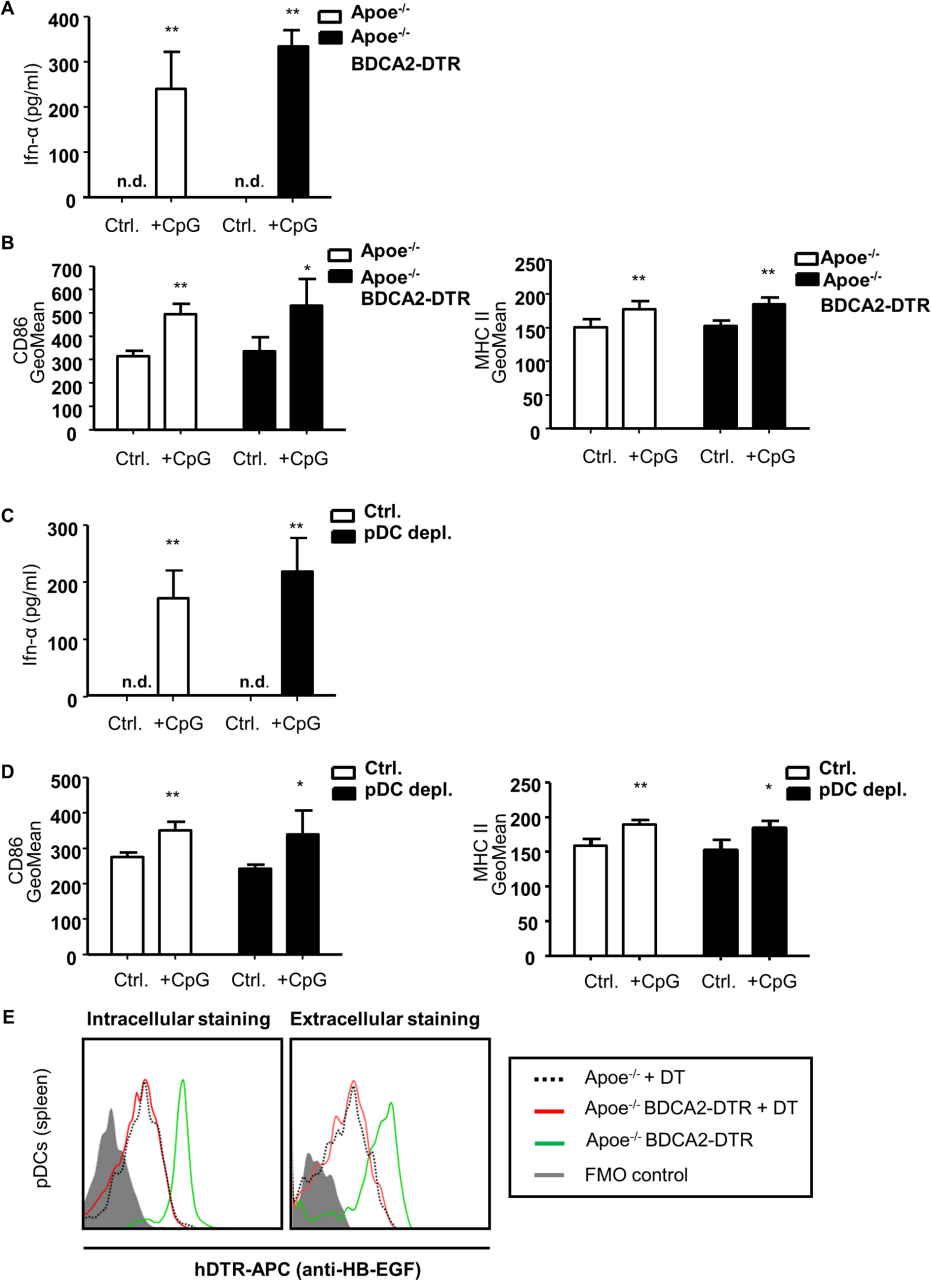
The BDCA2-DTR transgene does not influence pDC function in steady state and after 4 weeks HFD and DT injection. CD45^+^B220^+^440c^+^ pDCs were sorted from bone marrow of *Apoe* ^*-/-*^ and *Apoe* ^*-/-*^ *BDCA2-DTR* mice (A, B) fed normal chow or (C, D) receiving a HFD and 3 times weekly DT injection for 4 weeks. PDCs were cultured in RPMI1640 +/- CpG (5 μg/ml) for 12 hours at 37°C and (A, C) Ifn-α levels (pg/ml) were analyzed in cell culture supernatants and (B, D) expression of costimulatory molecules CD86 and MHCII on pDCs was measured with FACS. (E) Representative histogramms of intra- and extracellular hDTR (anti-HB-EGF) expression in *Apoe* ^*−/−*^ (dotted black), *Apoe* ^*−/−*^ *BDCA2-DTR mice* (pDC depleted, red) after 4 weeks of DT administration (i.p., 3 times weekly) and *Apoe* ^*−/−*^ *BDCA2-DTR* mice (green) without DT treatment. Graphs represent mean±SD; n = 5. Mann-Whitney test. *P<0.05

Since pDCs fully reoccur after three weeks (Fig 3) we were wondering if these pDCs would be fully functional again in comparison to their wild-type counterparts. To first exclude the possibility of a general impact of the BDCA2-DTR transgene on pDC function we compared Ifn-α release and upregulation of costimulatory molecules from sorted pDCs of *Apoe* ^*-/-*^ vs *Apoe* ^*-/-*^ *BDCA2-DTR* mice after CpG stimulation. Wild-type and transgenic pDCs significantly upregulated Ifn-α release as well as CD86 and MHCII expression 12 h after CpG stimulation in a comparable manner (Fig 4A and 4B). Further, reoccurring pDCs were sorted from *Apoe* ^*-/-*^ and *Apoe* ^*-/-*^ *BDCA2-DTR* mice after 4 weeks of HFD and DT treatment (3 times weekly), cultured and stimulated with CpG or left untreated. Again, Ifn-α release and CD86 as well as MHCII expression were significantly upregulated 12 h after stimulation compared to the untreated control, but there was no difference between wild type and transgenic pDCs (Fig 4C and 4D). To further investigate why reoccurring pDCs do not respond to DT treatment any longer we compared the intra- and extracellular DTR expression by flow cytometry analysis of splenic pDCs from *Apoe* ^*-/-*^ *BDCA2-DTR* mice before and after DT treatment. DT treated *Apoe* ^*-/-*^ mice were carried along as an independent control. As shown in Fig 4E, splenic pDCs from *Apoe* ^*-/-*^ *BDCA2-DTR* mice before DT treatment robustly express extra–and intracelluar DTR, however reoccurring pDCs after 4 weeks of DT administration have lost DTR expression and appear like the non-transgenic pDCs from *Apoe* ^*-/-*^ mice in histogram overlays (Fig 4E). Hence, we conclude that the insertion of the BDCA2-DTR transgene does not alter pDC function, but continuous DT treatment fosters expansion of DTR-negative but fully functional pDCs.

### Efficient one-week pDC depletion does not impact on plaque development of established atherosclerotic lesions

**Fig 5:**
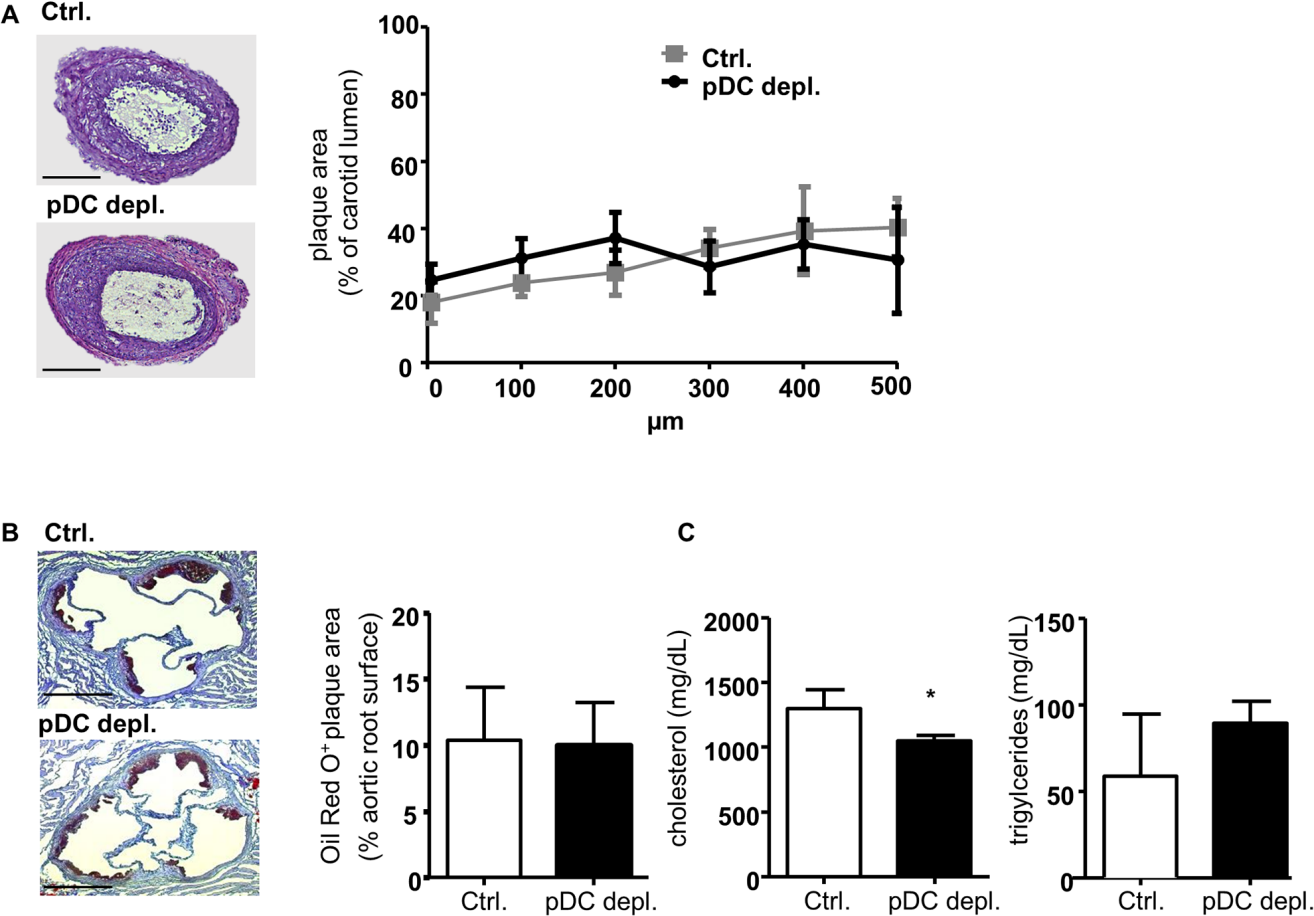
Efficient DT-induced pDC depletion does not influence aortic root and carotid lesion size after partial carotid artery ligation, but affects plasma Il-6 levels after 5 weeks of HFD and one week DT administration. (A) H&E staining of representative cryosections of the ligated left carotid artery (scale bar = 200 μm), quantification of plaque area (% of carotid lumen). (B) Aortic root lesions with Oil Red O^+^ area in % of aortic root surface (scale bar = 500 μm) and (C) plasma lipid levels of cholesterol and triglycerides (mg/dL) of *Apoe* ^*−/−*^ (control) and *Apoe* ^*−/−*^ *BDCA2 DTR* mice (pDC depleted) after 5 weeks of HFD and one week i.p. DT administration. Graphs represent mean±SD; n = 6 to n = 12. Mann-Whitney test. *P<0.05

**Fig 6:**
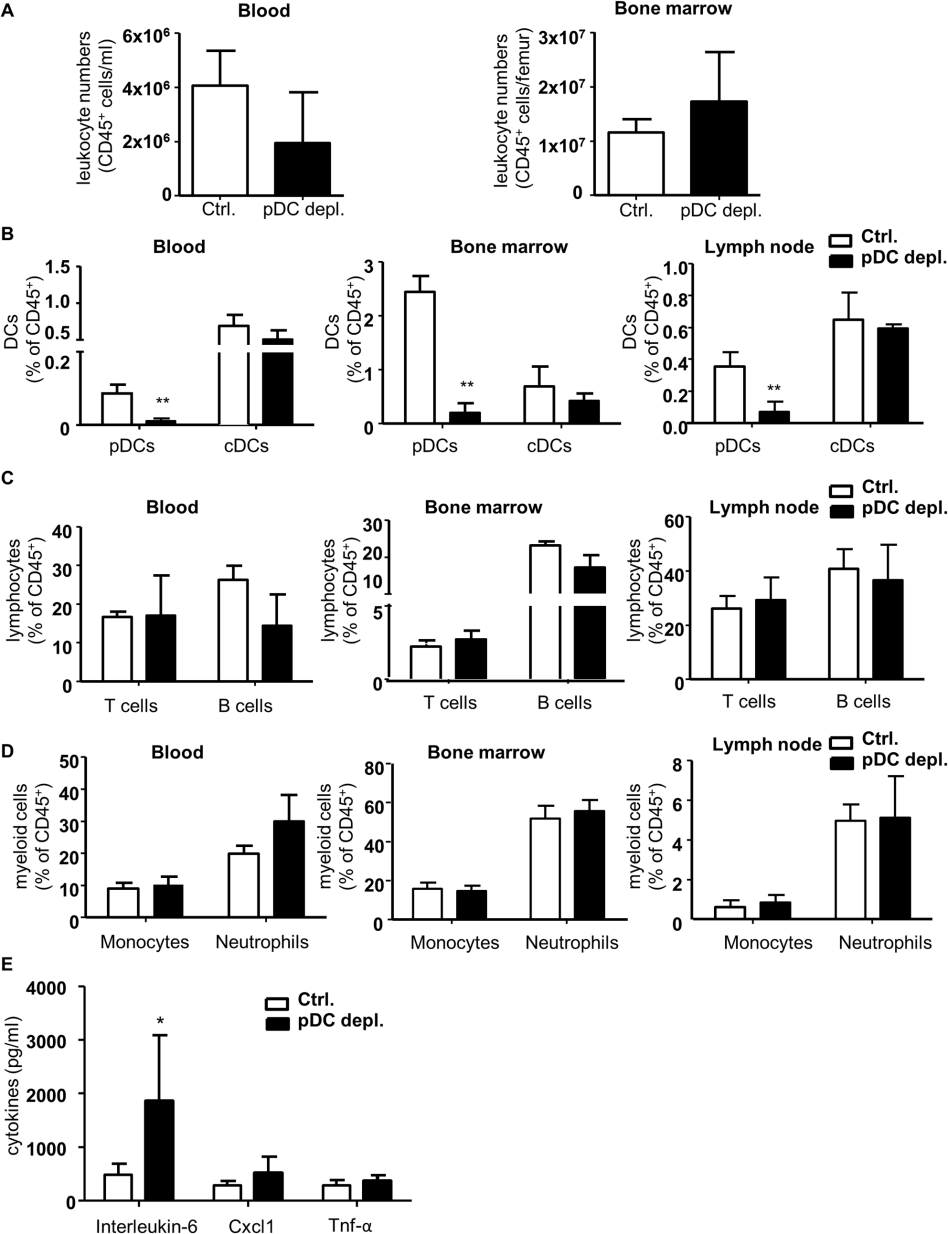
*Apoe* ^*−/−*^ *BDCA2-DTR* mice have no change in leukocyte numbers, but efficiently reduced pDC numbers after partial carotid artery ligation in combination with 5 weeks of HFD and one week of DT administration. (A) Absolute numbers of CD45^+^ leukocytes after 5 weeks HFD and one week DT administration in *Apoe* ^*−/−*^ (control) and *Apoe* ^*−/−*^ *BDCA2-DTR* mice (pDC depleted) in blood (cells/ml) and bone marrow (cells/femur). (B-D) Flow cytometry analysis of (B) dendritic cells (pDCs and cDCs), (C) lymphocytes (T and B cells) and (D) myeloid cells (neutrophils and monocytes) in blood, bone marrow and lymph node in *Apoe* ^*−/−*^ and *Apoe* ^*−/−*^ *BDCA2-DTR* mice after 5 weeks of HFD and one week DT administration. Gr1^+^CD115^-^ neutrophils, CD11b^+^CD115^+^ monocytes, CD3^+^ T cells, B220^+^ B cells, CD11c^+^MHCII^+^ cDCs and B220^+^mPDCA-1^+^440c^+^ pDC populations are shown as percentage of CD45^+^ leukocytes. (E) Plasma cytokine levels of Il-6, Cxcl1 and Tnf-α (pg/ml). Graphs represent mean±SD; n = 4 to n = 6. Mann-Whitney test *P<0.05 **P<0.01.

Based on the findings above we looked for another model allowing investigation of the role of pDCs in more advanced lesions while sticking to only one week depletion regimen. According to Nam et al. [24] partial carotid artery ligation causes endothelial dysfunction, flow disturbance and rapid atherosclerosis depicting advanced lesions already after 4 weeks. To examine if pDC depletion impacts on the development of established atherosclerotic plaques, we ligated the left carotid arteries of *Apoe* ^*-/-*^ and *Apoe* ^*-/-*^ *BDCA2-DTR* mice followed by HFD feeding for 5 weeks. DT was administered 3 times during the last week of HFD. Analysis of atherosclerotic lesions in the ligated carotid artery did not reveal any differences in lesion size (Fig 5A). Plaque development in aortic roots of these mice was also not significantly altered between the two groups (Fig 5B). Total cholesterol levels in blood were slightly diminished in *Apoe* ^*-/-*^ *BDCA2-DTR* mice, whereas triglyceride titers were not affected (Fig 5C). Moreover, flow cytometry analysis of blood, bone marrow and lymph nodes did not show any significant differences in leukocyte distribution, but just confirmed the successful depletion of pDCs in all three organs (Fig 6A–6D). Evaluating cytokine levels again, we saw a significant increase for Il-6, whereas Cxcl1 and Tnf-α were not significantly changed (Fig 6E). Again, no differences were detected for Ifn-α and Ccl2 (Fig Gb in S1 File).

Taken together, we conclude that efficient pDC depletion for one week is either not enough to directly influence advanced lesion development in the partial carotid ligation model, or pDCs do not interfere with progression or regression of established atherosclerotic lesions in this model.

### Efficient pDC depletion in indirect acute lung injury reduces early myeloid cell infiltration and lung damage

To exclude the possibility that breeding the *BDCA2-DTR* mice to *Apoe* ^*-/-*^ mice has a general impact on the functionality of this mouse model, we additionally analyzed the (performance of) *BDCA2-DTR* mice in an acute inflammation model and examined the role of pDCs in indirect acute lung injury (iALI). Earlier work by Crother et al.[27] and Venet et al.[28] already implicated a pro-inflammatory role of pDCs in iALI triggered by pulmonary bacterial infection. From various models of iALI that have been described, we decided for intravenous lipopolysaccharide (LPS) injection as injury trigger due to high reproducibility, sepsis context and a neutrophil inflammatory response, which is an important determinant of lung damage in early acute lung injury [29, 30].

**Fig 7:**
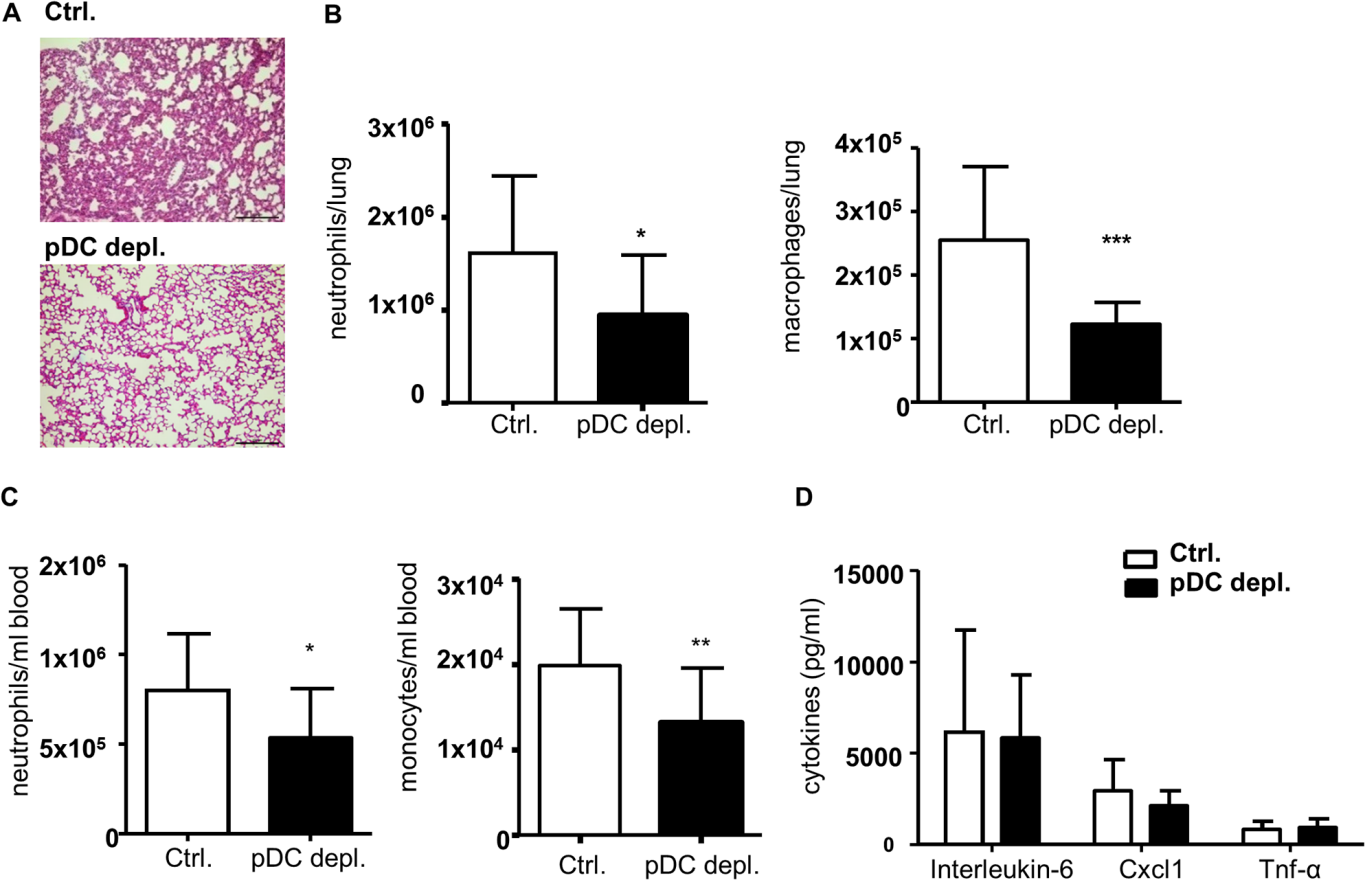
Efficient DT-induced pDC depletion reduces macrophage and neutrophil numbers in lungs and blood of *Apoe* ^*-/-*^ *BDCA2-DTR* mice after intravenous LPS administration in an iALI model. (A) H&E stained histological lung sections of *Apoe* ^*-/-*^ *BDCA2-DTR* mice after 24 hours after DT administration and 12 hours after i.v. LPS administration (100 μg/mouse) to iALI, (10x magnification and scale bars = 50 μm). (B) Flow cytometry analysis of CD45^+^Gr1^+^CD115^-^ neutrophils and F4/80^+^ macrophages in lungs (cells/lung) and (C) of CD45^+^Gr1^+^CD115^-^ neutrophils and CD45^+^CD11b^+^Gr1^-^CD115^+^ monocytes in blood (cells/ml) after iALI in pDC depleted *Apoe* ^*-/-*^ *BDCA2-DTR* mice. (D) Plasma cytokine levels of Il-6, Cxcl1 and Tnf-α (pg/ml). Graphs represent mean±SD; n = 9 to n = 16; Mann-Whitney test or unpaired t-test with Welch‘s correction *P<0.05 **P<0.01 ***P<0.001.

First, we depleted pDCs in *Apoe* ^*-/-*^ and *Apoe* ^*-/-*^ *BDCA2-DTR* mice with a single dose of DT (0.01 mg/kg) followed by intravenous injection of LPS 12 hours later. 24 hours after DT injection animals were sacrificed and examined. Efficient pDC depletion was confirmed by flow cytometry analysis of pDC numbers in blood, lymph node and bone marrow of LPS-treated *Apoe* ^*-/-*^ *BDCA2-DTR* mice (Fig Da-c in S1 file). Histological analysis of hematoxylin and eosin stained lung sections revealed reduced thickening of the alveolar spaces and intra-alveolar cell infiltrates in *Apoe* ^*-/-*^ *BDCA2-DTR* mice (Fig 7A). The latter was confirmed by flow cytometry analysis of enzymatically digested lung tissue, which revealed a significant reduction in the number of infiltrated leukocytes (Fig Fb in S1 file), particularly of neutrophils and macrophages (Fig 7B). This was mirrored by reduced numbers of neutrophils and monocytes in the blood (Fig 7C), however total leukocyte numbers in blood did not differ (Fig Fa in S1 file). Moreover, monocyte and neutrophil numbers in bone marrow were not altered in *Apoe* ^*-/-*^ *BDCA2-DTR* mice compared to controls (Fig Dc in S1 file). Further, CD45^+^ leukocytes in the bronchoalveolar lavage fluid (BALF) were only reduced by trend (Fig E in S1 file). Analysis of plasma cytokine levels did not reveal any differences for Il-6, Cxcl1 and Tnf-α (Fig 7D), nor for Ifn-α and Ccl2 (Fig Gc in S1 file).

In summary, using the BDCA2-DTR model in an acute inflammatory setting, we could confirm a pro-inflammatory role of pDCs in early immune responses in the context of LPS-induced iALI, indicating the usefulness of this mouse model to study the role of pDCs in acute inflammation.

## Discussion

This study aimed at dissecting the role of pDCs in advanced atherosclerotic lesions using *Apoe* ^*-/-*^ *BDCA2-DTR* mice, which allow specific depletion of pDCs using DT treatment.

However, despite ongoing DT injection three times weekly, complete depletion of pDCs could not be sustained for longer than one week. Further, DT treatment of *Apoe* ^*-/-*^ *BDCA2-DTR* mice neither affected atherogenesis (4 weeks HFD with 4 weeks DT treatment) nor atheroprogression (carotid ligation and 5 weeks HFD, with pDC depletion during the last week of HFD). Although this indicates that one-week pDC depletion does not affect atherosclerosis in these settings, these results do not argue against a role of pDCs in early or advanced lesions. They only underline the unsuitability of the *Apoe* ^*-/-*^ *BDCA2-DTR* mice for studying the involvement of pDCs in atherogenesis and-progression over extended time periods. In contrast, efficient pDC depletion in *Apoe* ^*-/-*^ *BDCA2-DTR* mice prior to LPS-induced iALI confirmed a pro-inflammatory role of pDCs in these settings, thus underlining the functionality of *Apoe* ^*-/-*^ *BDCA2-DTR* mice for studying pDCs in acute inflammation.

Several approaches have been made to generate mice allowing specific pDC depletion to limit and control side effects of prolonged cell ablation with antibodies. SiglecH-DTR mice have been created using the gene coding for sialic acid binding Ig-like lectin (Siglec)-H to control DTR expression, as SiglecH has been primarily described as a pDC-specific functional molecule. However, SiglecH-DTR transgenic mice are in fact deficient in SiglecH expression owing to interference with its transcriptional or posttranscriptional machinery [31].Furthermore, propagated SiglecH-expression is not restricted to pDCs and was reported to be also expressed by marginal zone macrophages.[32] Thus, SiglecH-DTR mice have to be considered with caution when examining the role pDCs *in vivo* and were therefore excluded from our study. Instead we decided for the BDCA2-DTR model, which was first published by Swiecky et al. to examine pDC function in a model of murine cytomegalovirus infection. In this study the authors only examined short term depletion of pDCs for three days, but do also state that pDCs could be depleted for longer periods with repeated DT administration, without showing data or specifying on how long depletion was possible.[23] Similarly, Swiecky et al. also examined how type IFN-I of different sources control pDC numbers in (chronic) viral infections (Hepatitis B and C, HIV) applying again the BDCA2-DTR model. Still, only short term depletion (single DT injection) experiments were conducted. [33] Yet, another study from the same group evaluated the impact of early transient pDC depletion in a murine model of SLE (*BXSB*.*MpJ* bred with *BDCA2-DTR* mice). Here pDCs were depleted over three weeks (DT i.p. every 3–4 days for 3 weeks) and depletion efficiency after three weeks was confirmed showing representative dot plots of spleen cells with a pDC depletion efficacy of more than 95%. However the authors show representative average depletion efficiencies in spleen only and do not detail on pDC numbers in other organs.[25] Notably, in our hands splenic pDCs numbers were already restored to 50% of control level after 2 weeks.

A number of other studies have also used the BDCA2-DTR model to examine pDC function in various pathophysiological contexts. Nevertheless, pDC depletion was not continued over more than two weeks. For example Glitzner et al. evaluated the impact of pDCs and Langerhans cells in the initiation and progression of psoriasis (mouse model: *Jun* ^*f/f*^ *JunB* ^*f/f*^ *K5Cre-ERT* bred to *BDCA2-DTR* mice). Here, repetitive DT injections were administered up to 15 days and flow cytometry analysis of spleen and dermis at day 15 depicted over 90% reduction of pDCs in both organs.[26] Further, successful pDC ablation in spleen and lymph node of *BDCA2-DTR* mice was also reported in a mouse melanoma model, however in this study cells were only depleted for 6 days.[34] In summary, data sufficiently supporting sustained pDC depletion in *BDCA2-DTR* mice for more than two weeks have not yet been published. Furthermore, one can also not exclude that breeding *BDCA2-DTR* mice to different mouse models may be associated with differential pDC depletion efficiencies due to strain-dependent BDCA2 expression levels. Although the *BDCA2* gene is normally not expressed in the mouse genome, transcriptional regulation of the human BDCA2 promoter in mice could be associated with small strain-dependent differences, and could cause insufficient or even off-target expression of DTR in *BDCA2-DTR* mice depending on the mouse strain. Conclusively, variable BDCA2 expression in different strains could account as one explanation for different depletion efficiencies e.g. comparing flow cytometry analysis in the spleen between our study and results by Glitzner et al. Still, sustained pDC depletion over more than 2 weeks awaits to be proven in any of the models mentioned above.

Moreover, studies examining atherosclerosis in DTR models over more than 2 weeks are in general scarce. Causative could be that promoters of more ubiquitously and hence less specifically expressed markers like e.g. CD11c or CD11b in combination with DTR do tolerate repetitive DT injections only if bone marrow chimeras are generated, assuring that none-immune cells remain of non-transgenic origin and are therefore not able to express the DTR.[35] Hence, off-target DTR expression seems to be a general problem[35] and its likeliness probably increases with prolonged DT treatment. Nevertheless, Stoneman et al. showed a significant reduction in atherosclerotic lesion development in *CD11b-DTR* bone marrow chimeras applying DT for 10 weeks. These changes in lesion size were attributed to a significant reduction in circulating monocyte counts and macrophage numbers in the plaque. If neutrophils were also affected by DT treatment was not addressed in detail.[36] Further, bone marrow of *DEREG* mice expressing DTR under the control of FoxP3, the transcriptional regulator of regulatory T cell (Treg) development, was transplanted in low density lipoprotein receptor deficient mice (*Ldlr* ^*-/-*^) and repetitive DT injections were administered for 8 weeks.[37] Subsequent analysis of lesion growth revealed significantly increased plaque development in Treg-depleted animals. Ablation efficiency was monitored in blood after 10, 30 and 60 days of DT injections and surprisingly total Treg numbers were not altered between the control and the DT treated group. However, differentiating between GFP^+^ Treg (donor-derived) and GFP^-^ Treg (of recipient origin) displayed a significant reduction of GFP^+^ Treg. Still, GFP^+^ Treg depletion efficiency decreased over time from over 90% (10 days) to 50% (60 days). Remarkably, in parallel the frequencies of GFP^-^ Treg increased over time, which the authors explain by expansion of non-functional recipient Tregs, as these cells could not compensate the observed effects on atherosclerosis.[37] Yet, another very recent study shows that DT treatment of *DEREG* mice induces emergence of a donor-derived Treg population that has lost DTR expression and are therefore DT resistant.[38] Emerging Tregs in *DEREG* mice were also described before [39, 40], but the mechanisms behind remain elusive. Notably, we could not detect DTR expression in/on reoccurring pDCs after 4 weeks DT treatment, but found these cells to be fully functional again. Taken together, these data suggest that emergence of targeted cell populations that have lost DTR expression are an important explanation for insufficient cell depletion in many DTR models. The latter might be due to selective expansion of DTR-negative cells induced by a continuous ‘feedback loop’ of the sensor branch points controlling cell homeostasis.

To confirm functionality of the BDCA2-DTR mouse model before potential onset of reduced DTR expression upon long-term DT treatment, we included an LPS-induced iALI model, as recent studies have evaluated the role of pDCs in bacterial infection triggering ALI. [27, 28] In a model of iALI, pDC depletion enhanced the number of infiltrating monocytes and neutrophils after 24 h [28], instead chlamydia pneumonia-infected mice displayed delayed recruitment of monocytes and neutrophils if pDC were depleted (either with a depletion antibody or in *BDCA2-DTR* mice) [27]. Inducing iALI in *BDCA2-DTR* mice by LPS injection, we did also see diminished macrophage and neutrophil accumulation in the lung in the absence of pDCs, confirming observations of Crother et al.[27]. As this was mirrored by reduced numbers of neutrophils and monocytes in the blood, our data suggest a diminished systemic mobilization of these cells upon iALI of mice lacking pDCs. However, one can also not exclude that changes in circulating monocyte and neutrophil numbers could be attributed to changes in life span or enhanced clearance. In conclusion, using the BDCA2-DTR mouse model, we confirm a pro-inflammatory role of pDCs in iALI by inducing enhanced levels of myeloid cells in blood and injured lung tissue. This underlines the usefulness of the BDCA2-DTR mouse model to study pDCs in acute inflammatory settings.

Altogether, our study reveals for the first time an unsuitability of *BDCA2-DTR* mice for studying the involvement of pDCs in disease models over extended time periods, whereas it underlines its functionality for studying pDCs in acute inflammatory settings.

## Supporting Information

S1 ARRIVE Checklist NC3Rs ARRIVE Guidelines Checklist. (DOCX)

S1 File Supplementary information Mandl. et al.
(PDF)
