# Supplementary material for: Evaluation of the BDCA2-DTR Transgenic Mouse Model in Chronic and Acute Inflammation
Source: PLoS One. 2015 Aug 7;10(8):e0134176. doi: 10.1371/journal.pone.0134176 (PMC4529211; doi:10.1371/journal.pone.0134176)

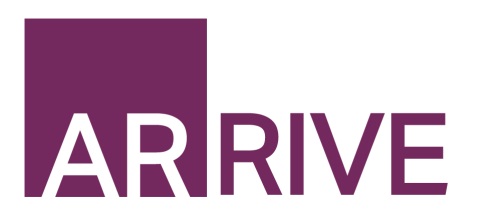


The ARRIVE Guidelines Checklist

Animal Research: Reporting In Vivo Experiments

Carol Kilkenny^1^, William J Browne^2^, Innes C Cuthill^3^, Michael Emerson^4^ and Douglas G Altman^5^

*^1^The National Centre for the Replacement, Refinement and Reduction of Animals in Research, London, UK, ^2^School of Veterinary Science, University of Bristol, Bristol, UK, ^3^School of Biological Sciences, University of Bristol, Bristol, UK, ^4^National Heart and Lung Institute, Imperial College London, UK, ^5^Centre for Statistics in Medicine, University of Oxford, Oxford, UK.*

|  | | ITEM | RECOMMENDATION | Section/ Paragraph |
| --- | --- | --- | --- | --- |
| 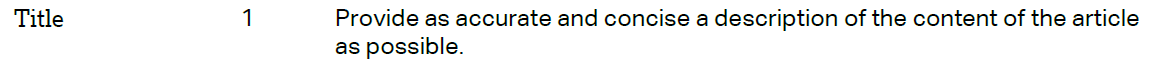 | | | Title |  |
| 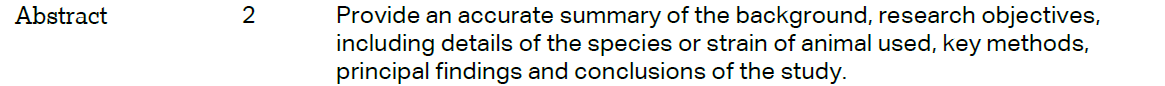 | | | Abstract |  |
| INTRODUCTION | | |  |  |
| 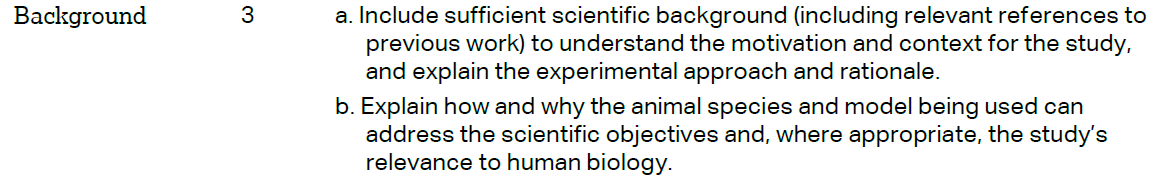 | | | Introduction  “Background and Aims” |  |
| 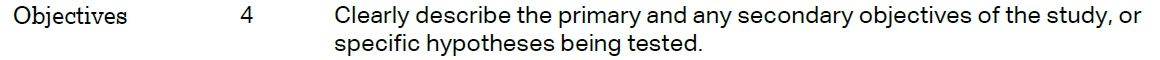 | | | Introduction  “Background and Aims” |  |
| METHODS | | |  |  |
| 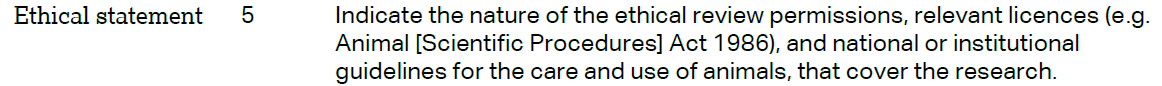 | | | Methods Paragraph1 |  |
| 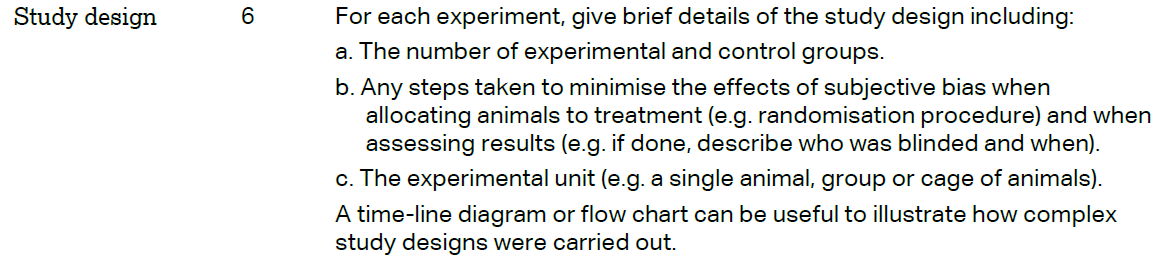 | | | Methods Paragraph2+3  Results “Figure legends”  Figure 3A |  |
| 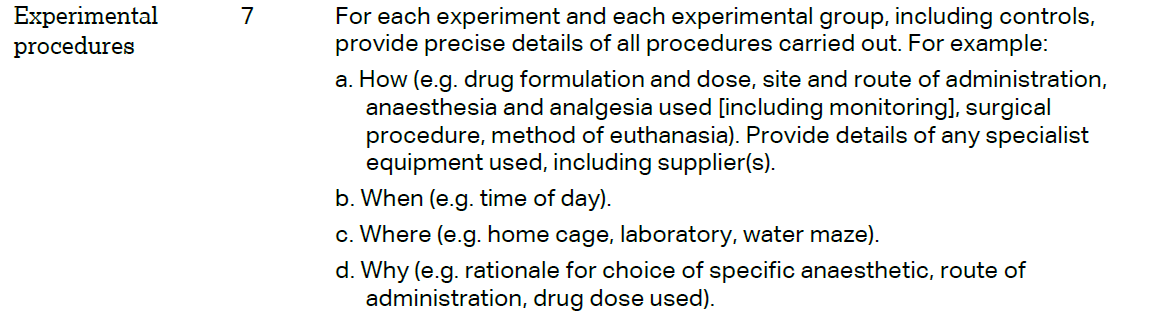 | | | Methods  Paragraph 1-4 |  |
| 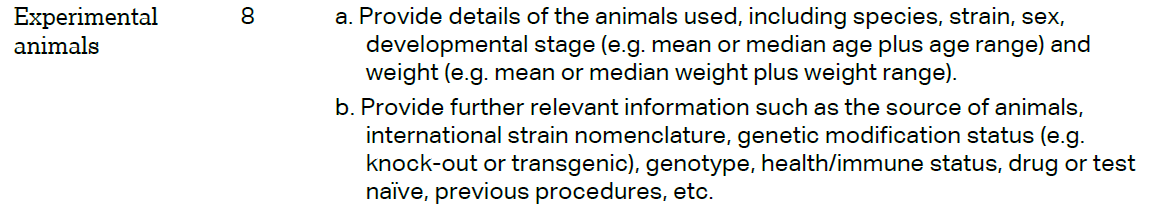 | | | Methods Paragraph1 |  |

The ARRIVE guidelines. Originally published in *PLoS Biology*, June 2010^1^

| 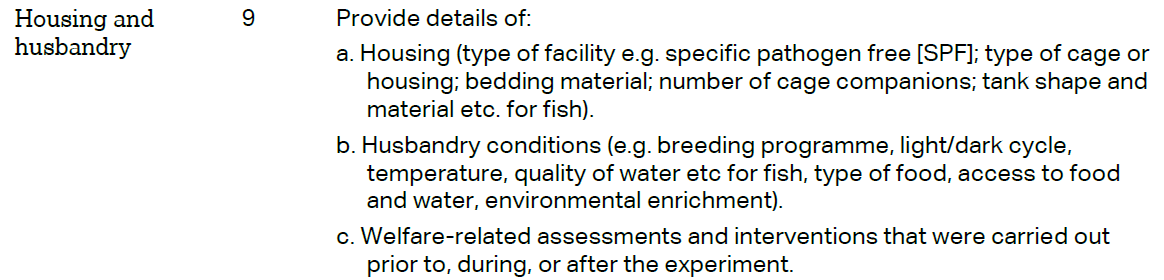 | Methods Paragraph1 | |
| --- | --- | --- |
| 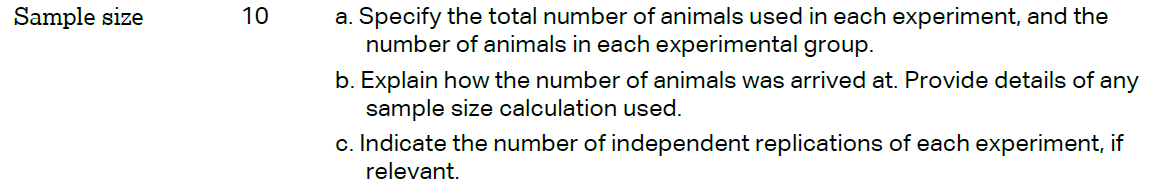 | Methods Paragraph 1 and “Figure legends” | |
| 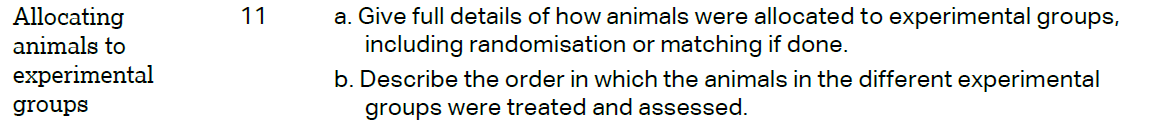 | Mice were allocated to experimental groups according to their genotype  Treatment see Methods  “in vivo depletion of pDCs” and “LPS-induced indirect acute lung injury” | |
| 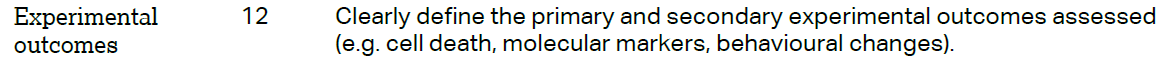 | Atherosclerotic lesions size, pDC depletion efficiency, lung damage, leukocyte numbers | |
| 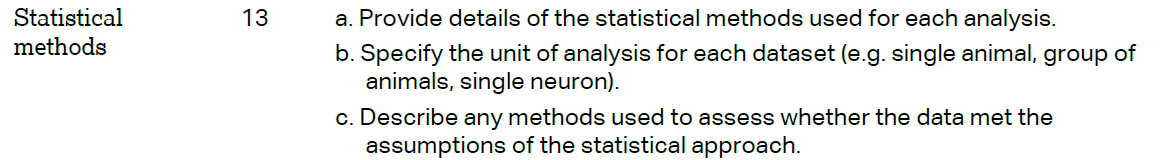 | Methods  “Statistics” | |
| RESULTS |  | |
| 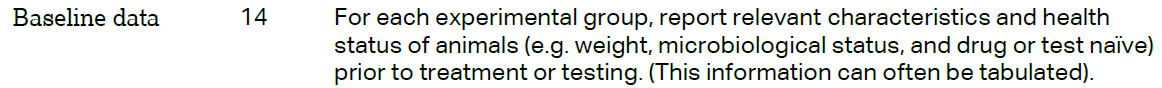 | Methods  Paragraph 1 | |
| 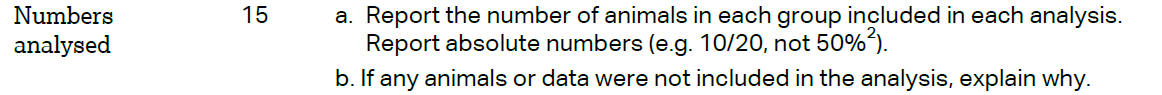 | See “Figure legends”  No animals were excluded | |
| 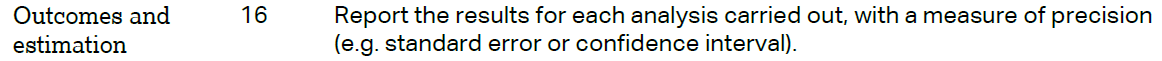 | Results  Paragraph 1 -5 and figures | |
| 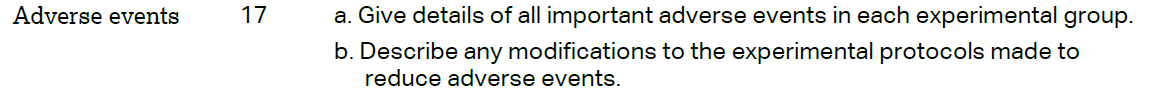 | No adverse events occurred  Methods Paragraph 1 | |
| DISCUSSION |  | |
| 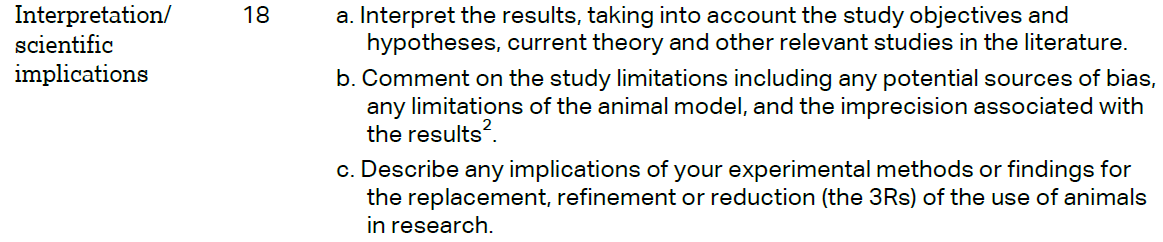 | Discussion  (throughout) | |
| 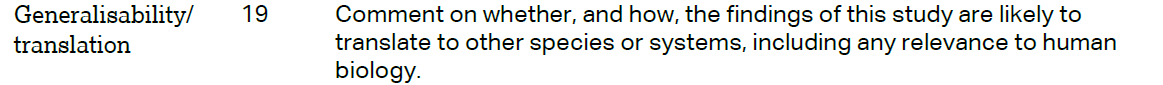 | Does not apply | |
| 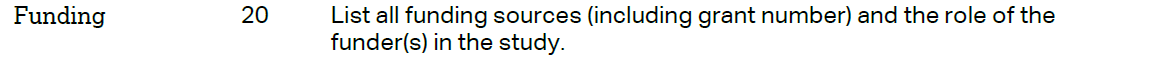 | | SFB1054, B4  SFB1123,  A1, A6, B5  LMUexcellent  German Centre for Cardiovascular Research (MHA VD1.2)  The funders had no role in study design, data collection and analysis, decision to publish, or preparation of the manuscript. |


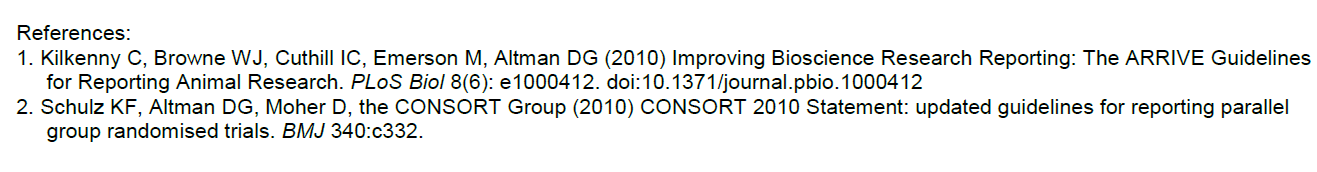

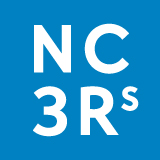

Supplement: S1 ARRIVE Checklist — (DOCX) [file pone.0134176.s001.docx]
