# Supplementary material for: Evaluation of the BDCA2-DTR Transgenic Mouse Model in Chronic and Acute Inflammation
Source: PLoS One. 2015 Aug 7;10(8):e0134176. doi: 10.1371/journal.pone.0134176 (PMC4529211; doi:10.1371/journal.pone.0134176)
Supplement: S1 File — et al. (PDF) [file pone.0134176.s002.pdf]

Supplementary Information Mandl et al.

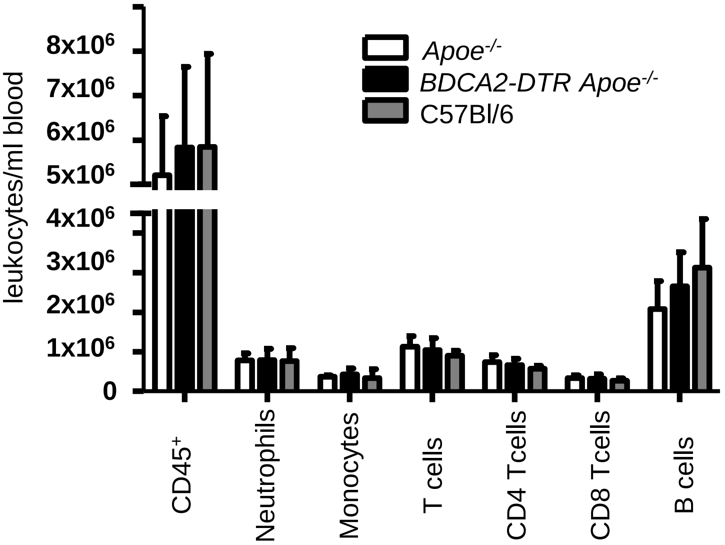

Figure A Flow cytometry analysis of leukocyte populations in blood of *Apoe*$^{-/-}$, *Apoe*$^{-/-}$ *BDCA2 DTR* and C57Bl/6 mice. CD45^{+}Gr1^{+}CD115^{-} neutrophils, CD45^{+}CD11b^{+}CD115^{+} monocytes, CD3^{+} CD4^{+} or CD8^{+} T cells and B220^{+} B-cells are shown as absolute numbers (cells/ml) in peripheral blood.

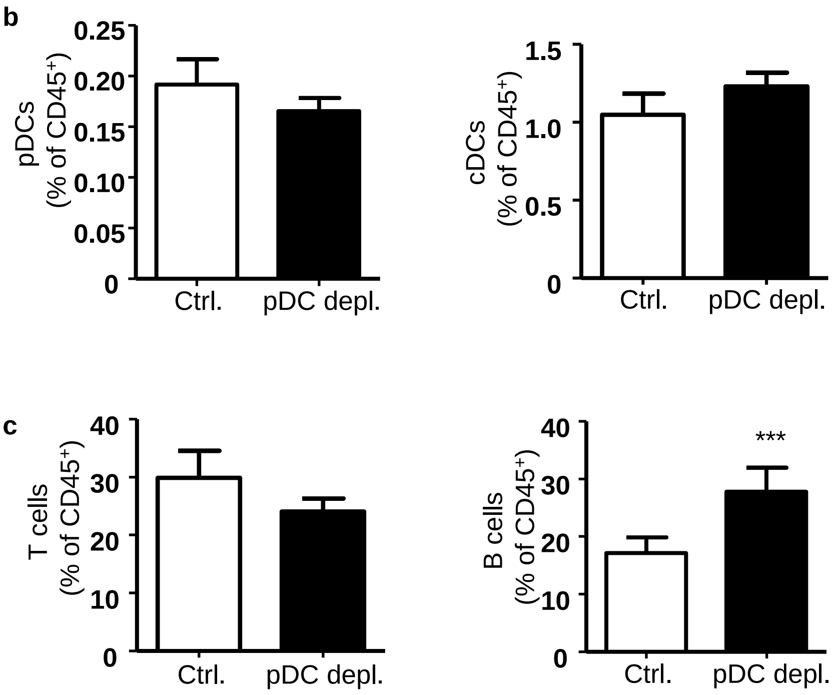

Figure B Flow cytometry analysis of axillary lymph nodes (LN) in *Apoe*$^{-/-}$ (control) and *Apoe*$^{-/-}$ *BDCA2-DTR* (pDC depleted) mice after 4 weeks HFD and DT administration. **(a)** Dendritic cell subsets (pDCs and cDCs) and **(b)** lymphocytes ($CD3^+$ T cells and $B220^+$ B cells) are shown as percentage of $CD45^+$ cells in axillary lymph nodes. Graphs represent mean±SD; n=6 to n=10. Mann-Whitney test ****P*<0.001.

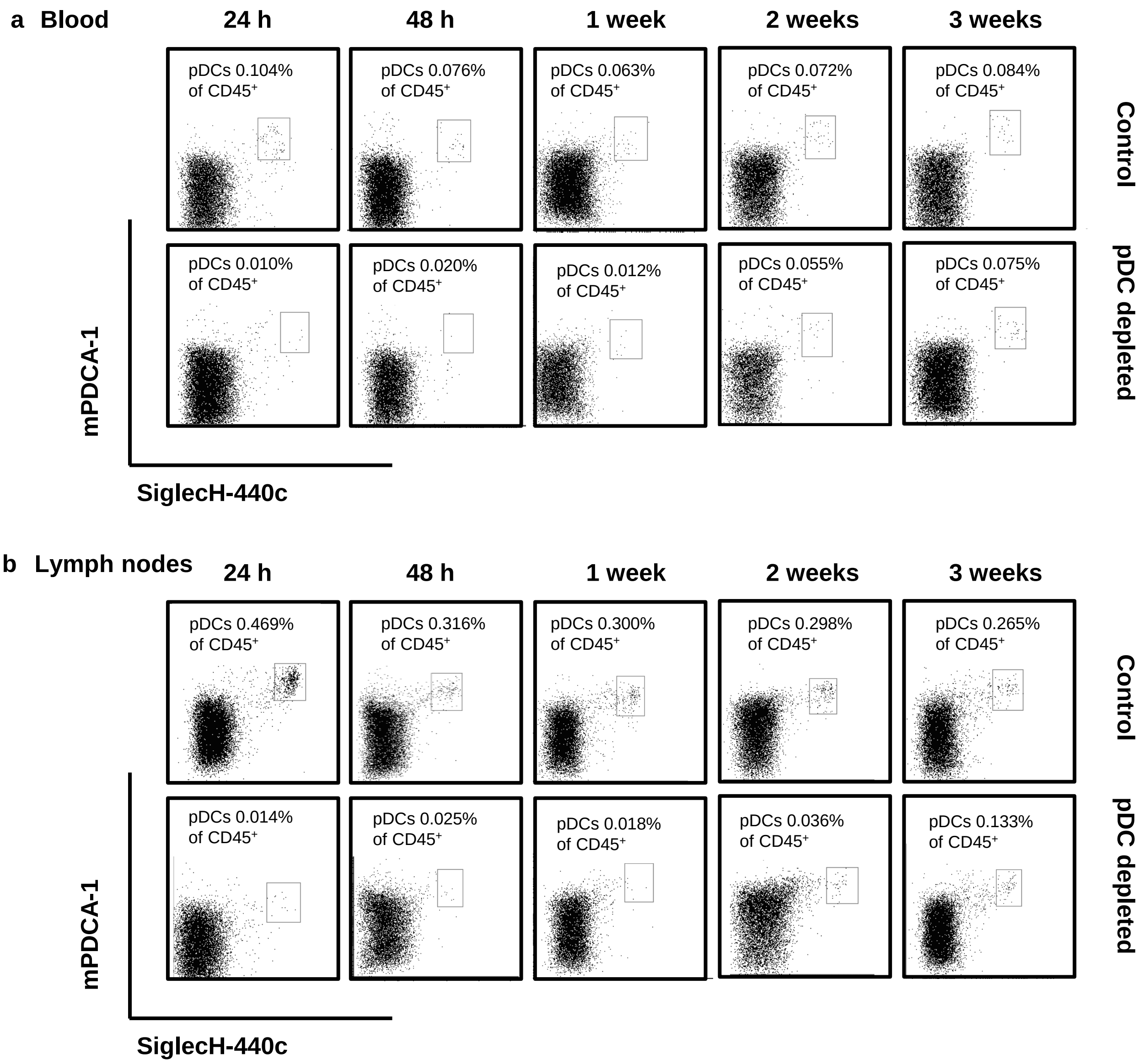

Figure C Representative dot plots depicting the depletion efficiency of pDCs in blood **(a)** and axillary lymph nodes **(b)** after i.p. DT administration in *Apoe*$^{-/-}$ (control) and *Apoe*$^{-/-}$ *BDCA2-DTR* mice (pDC depleted). Representative dot plots show B220$^+$mPDCA-1$^+$440c$^+$ pDC populations as percentage of CD45$^+$ cells in blood 24 and 48 hours, one week, two weeks and three weeks after continous DT administration.

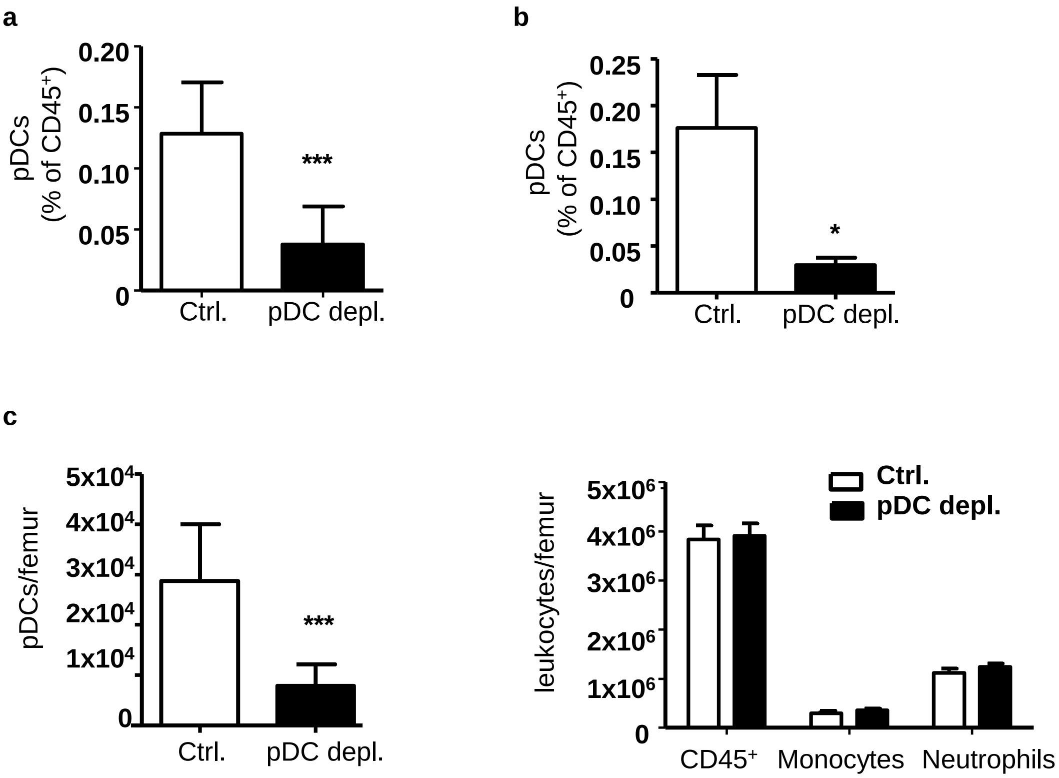

Figure D pDC and leukocyte frequencies in *Apoe*$^{-/-}$ *BDCA2-DTR* mice compared to *Apoe*$^{-/-}$ mice 12 hours after iALI and 24 hours after DT application in **(a)** blood (% of CD45^{+} cells), **(b)** axillary lymph nodes (% of CD45^{+} cells) and **(c)** bone marrow (cells/femur). Graphs represent mean±SD; n=9 to n=16. Mann-Whitney test or unpaired t-test with Welch‘s correction **P*<0.05 ***P*<0.01.

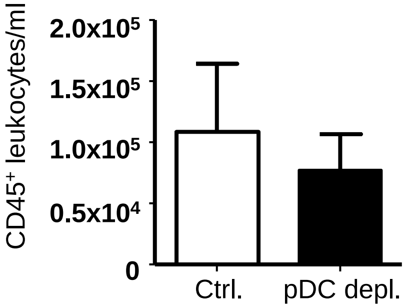

Figure E Flow cytometry analysis of CD45$^+$ leukocytes (cells/ml) in bronchoalveolar lavages (BALF) after iALI in *Apoe*$^{-/-}$*BDCA2-DTR* mice compared to *Apoe*$^{-/-}$ controls. Graphs represent mean±SD; n=4 to n=6.

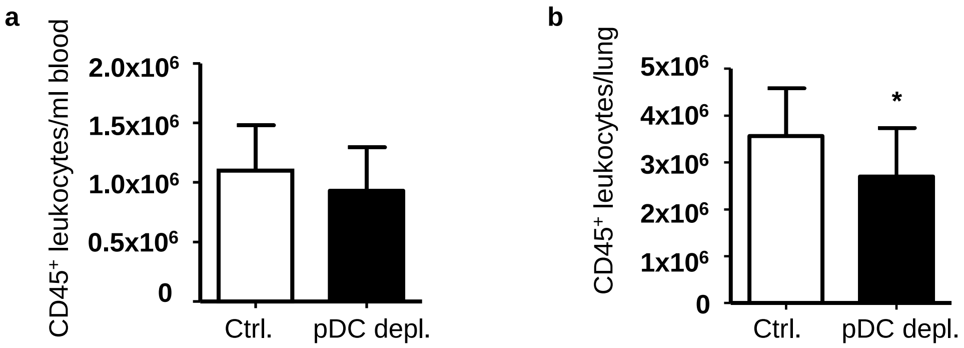

Figure F Flow cytometry analysis of $CD45^{+}$ leukocytes in **(a)** blood (cells/ml) and **(b)** lungs (cells/lung) after iALI in *Apoe*$^{-/-}$ *BDCA2-DTR* mice compared to non-depleted control mice. Graphs represent mean±SD; n=9-16. Unpaired t-test with Welch‘s correction *P<0.05.

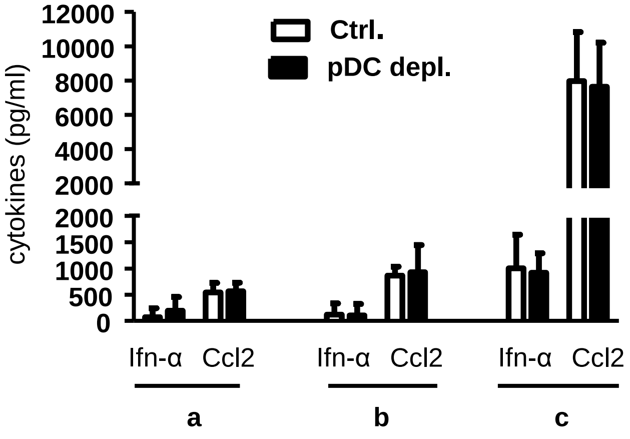

Figure G Plasma cytokine levels of Ifn-α and Ccl2 (pg/ml) for **(a)** atherosclerosis (4 weeks HFD) **(b)** carotid artery ligation and **(c)** iAli experiments in *Apoe*$^{-/-}$ *BDCA2-DTR* mice compared to non-depleted *Apoe*$^{-/-}$ control mice. Graphs represent mean±SD; n=6-16.
